# Potential Economic and Clinical Implications of Multi-Dose Intravenous Acetaminophen After Robotic-Assisted Prostatectomy: A Secondary Descriptive Analysis of Publicly Available Phase IV Trial Data (NCT02369211)

**DOI:** 10.3390/healthcare14101367

**Published:** 2026-05-16

**Authors:** Majed Ahmed Algarni

**Affiliations:** Department of Clinical Pharmacy, College of Pharmacy, Taif University, P.O. Box 11099, Taif 21944, Saudi Arabia; m.alqarni@tu.edu.sa

**Keywords:** intravenous acetaminophen, multimodal analgesia, opioid-sparing effect, robotic-assisted laparoscopic prostatectomy (RALP), postoperative recovery, hospital length of stay (LOS)

## Abstract

**Introduction:** This study evaluated the implications of intravenous acetaminophen (Ofirmev) on perioperative outcomes in patients undergoing robotic-assisted laparoscopic prostatectomy (RALP) for prostate cancer. The primary objective was to determine whether adding IV acetaminophen to standard analgesia could reduce hospital length of stay (LOS), pain intensity, and opioid use compared with placebo. **Methods:** This study was conducted as a secondary descriptive analysis of publicly available aggregate results from a previously completed randomized, double-blind, placebo-controlled Phase IV trial (NCT02369211). No individual patient-level data were accessed, and no new independent statistical analyses were performed. Eighty-six male patients were randomly assigned to receive either 1 g IV acetaminophen or saline placebo every six hours for four doses during the perioperative period. Primary endpoints were hospital and post-anesthesia care unit (PACU) LOS; secondary endpoints included postoperative pain scores and opioid consumption (morphine milligram equivalents). **Results:** Baseline characteristics were similar between groups (n = 43 each). Mean PACU stay was slightly shorter with IV acetaminophen (124 ± 58 min) than placebo (132 ± 63 min; not significant). Median hospital LOS was 0.81 days versus 0.82 days (*p* = 0.006), a statistically significant difference reported in the original trial dataset, although the absolute difference was clinically minimal. Pain scores and opioid requirements were lower with IV acetaminophen but not significantly different. No adverse events occurred in either group. **Conclusions:** IV acetaminophen was safe and well tolerated as part of multimodal analgesia for RALP. Although pain scores and opioid use numerically favored IV acetaminophen, these differences were not statistically significant. The reported difference in hospital LOS was statistically significant in the original trial record but clinically minimal; therefore, the findings should be interpreted as exploratory and hypothesis-generating regarding potential operational and economic implications.

## 1. Introduction

Intravenous acetaminophen (IV acetaminophen) is a complex therapeutic formulation affecting postoperative pain management and recovery outcomes in clinical practice [1]. Like other multimodal analgesic approaches, its main function is to reduce pain while minimizing the use of opioid medications and their associated side effects [2]. Patients treated with multi-dose IV acetaminophen often experience reduced pain intensity, lower opioid consumption, and faster recovery after surgery [1,2,3]. Some patients report better tolerance, less nausea, and an overall improvement in comfort and mobility during the postoperative period. Additional benefits can also develop, such as shorter stays in recovery units, reduced need for antiemetic drugs, and greater satisfaction with pain control [4]. From an economic perspective, the condition is reported to be favorable when IV acetaminophen use leads to lower indirect healthcare costs through faster discharge and fewer complications [5,6].

IV acetaminophen is an effective non-opioid analgesic that acts centrally by inhibiting prostaglandin synthesis and modulating serotonergic pathways in the brain and spinal cord [7,8]. Unlike oral or rectal forms, the IV route ensures rapid absorption, consistent plasma concentrations, and a predictable therapeutic response, making it ideal for patients undergoing surgery or those unable to tolerate oral intake [7,8]. In clinical practice, OFIRMEV is commonly administered as part of multimodal pain management to achieve adequate pain control while reducing the need for opioids [9]. Several studies have demonstrated that multi-dose administration of IV acetaminophen may reduce pain scores and decrease opioid consumption within the first 24 h after surgery, though the magnitude of effect and the consistency across surgical settings vary [10,11,12]. These outcomes are linked to enhanced patient comfort, accelerated mobilization, and a decreased incidence of opioid-related adverse effects such as nausea, vomiting, and respiratory depression [10,11,12].

The recent developments in postoperative care have been more geared towards formulating pain management approaches that not only enhance patient comfort but also maximize the efficiency of the hospital as well as minimize healthcare expenditures [13,14]. In this regard, IV acetaminophen (OFIRMEV) has become an essential pharmacological agent in multimodal analgesia [9]. It is well recognized for possessing the ability to deliver effective analgesia without inducing adverse effects that are associated with opioids or nonsteroidal anti-inflammatory drugs (NSAIDs) [15,16]. The economic outcomes of OFIRMEV involve the reduction in opioid utilization, the duration of recovery room stays and hospitalization, and the administration of adjunctive medications such as antiemetics or sedatives. All these improvements can lead to a decrease in the length of hospitalization and indirect healthcare costs [17].

The primary objective of OFIRMEV therapy is to provide rapid and safe analgesia while potentially improving perioperative efficiency without compromising patient satisfaction or increasing postoperative complications. Although its clinical benefits are well established, continued research is needed to determine the most cost-effective dosing regimens and to assess its overall impact on hospital resource utilization across different surgical specialties.

Despite growing evidence supporting intravenous acetaminophen as part of multimodal analgesia, limited literature has specifically examined its potential operational and economic implications in robotic-assisted laparoscopic prostatectomy. In addition, most available economic evidence comes from heterogeneous surgical populations or retrospective database studies, leaving uncertainty regarding the interpretation of publicly available randomized trial data in minimally invasive urologic surgery.

The aim of this study was to descriptively evaluate publicly available aggregate results from a completed Phase IV randomized trial of multi-dose IV acetaminophen in patients undergoing RALP, with emphasis on hospital LOS, PACU LOS, pain scores, opioid consumption, safety outcomes, and potential operational and economic implications.

## 2. Methods

### 2.1. Study Design and Data Source

This study represents a secondary descriptive analysis of publicly available results from the registered Phase IV randomized, double-blind, placebo-controlled clinical trial NCT02369211 evaluating multi-dose intravenous acetaminophen following robotic-assisted laparoscopic prostatectomy (RALP) [9]. The authors did not conduct the original randomized trial and utilized only publicly accessible aggregate data and registry materials available through ClinicalTrials.gov and associated publicly available protocol documents.

According to the registered study protocol, participants in the original trial were randomly assigned in a double-blind manner to receive either 1 g intravenous acetaminophen or intravenous saline placebo within 15 min following induction of anesthesia and prior to surgical incision, with repeat doses administered every 6 h for a total of four perioperative doses.

The primary endpoints of the original trial were post-anesthesia care unit (PACU) length of stay and hospital length of stay (LOS). Secondary endpoints included postoperative pain intensity assessed using the Visual Analog Scale (VAS) and postoperative opioid consumption expressed as morphine milligram equivalents (MME).

The present study aimed to descriptively evaluate the reported clinical and potential economic implications associated with intravenous acetaminophen as part of multimodal perioperative analgesia in patients undergoing RALP. Because the present work was conducted as a secondary descriptive analysis of a single publicly available clinical trial rather than a systematic review or meta-analysis, PRISMA methodology and prospective review registration were not applicable.

### 2.2. Variables and Outcomes Evaluated

Variables evaluated in the present analysis included perioperative opioid consumption, post-anesthesia care unit (PACU) length of stay, hospital length of stay, postoperative recovery outcomes, and safety-related findings as reported in the original trial materials. Information regarding trial characteristics, participant numbers, dosing schedules, and reported statistical outcomes was also extracted where publicly available.

According to the original study protocol, a sample size of 44 patients per group was estimated to provide 90% power to detect a 12 min difference in PACU length of stay at a significance level of 0.05.

### 2.3. Data Interpretation Approach

Publicly reported clinical and perioperative outcomes from the original Phase IV randomized trial were descriptively evaluated to explore their potential clinical and economic implications. Reported statistical outcomes, including *p* values and confidence intervals where available, originated from the original trial analyses. The present study did not involve independent statistical reanalysis of individual-level patient data and instead focused on descriptive clinical and economic interpretation of the published findings.

### 2.4. Ethical Considerations

The present study utilized publicly available anonymized trial registry data and did not involve direct human participant recruitment, identifiable patient information, or access to individual-level private data. Therefore, additional institutional ethical approval was not required.

## 3. Results

This section presents a descriptive summary of publicly reported aggregate results from a Phase IV randomized, double-blind, placebo-controlled clinical trial evaluating perioperative outcomes associated with intravenous acetaminophen use. No new analyses were independently conducted by the authors.

All results and numerical data were obtained from the publicly accessible record on ClinicalTrials.gov (Study ID: NCT02369211) [18].

Although 86 participants were initially enrolled, two in the placebo group did not complete the study, resulting in 84 participants included in the final analysis (43 in the IV acetaminophen group and 41 in the placebo group). The trial was initially registered on 17 February 2015, and the verified results were first posted on 11 September 2019, following a detailed quality control review by the National Library of Medicine. The differing participant numbers across outcome analyses reflect incomplete or unavailable registry-reported outcome data for some participants. This may introduce attrition bias and could affect internal comparability between groups.

### 3.1. Baseline Characteristics

When categorized by age range, it was observed that 35.7% of all participants were between 18 and 65 years old, while 64.3% were 65 years or older (see Table 1). None of the enrolled participants was younger than 18 years, which is consistent with the study’s inclusion criteria that limited participation to adults and older adults. This demographic distribution suggests that the majority of individuals represented a population more susceptible to postoperative complications and prolonged recovery, which makes the evaluation of economic interventions especially relevant.

### 3.2. Primary Outcomes

#### 3.2.1. Post-Anesthesia Care Unit (PACU) Length of Stay

Following surgery, each participant was transferred to the PACU, where the duration of stay ranged from 30 to 240 min. Patients who received IV acetaminophen had a mean recovery time of 124 ± 58.26 min, while those who received placebo had a slightly longer duration of 132 ± 62.58 min. Although this difference was modest, it indicates a numerical trend toward shorter PACU stay among patients treated with acetaminophen; however, the difference was not statistically significant and should not be interpreted as evidence of clinically meaningful improvement in postoperative recovery efficiency.

The number of participants analyzed for PACU length of stay was different between the groups because some patients’ PACU records were missing or incomplete. As a result, data were available for only 34 participants in the IV acetaminophen group and 41 in the placebo group [18] (see Table 2).

#### 3.2.2. Hospital Length of Stay (LOS)

The median hospital LOS was 0.81 days (95% CI: 0.71–0.90) in the acetaminophen group and 0.82 days (95% CI: 0.66–0.95) in the placebo group. The hospital LOS data was available for 34 participants in the IV acetaminophen group and 41 participants in the placebo group because some patients had missing or incomplete hospital records. These findings indicate that the use of IV acetaminophen was not associated with an increase in hospital length of stay, an important consideration in economic assessments. Although statistical significance was reported in the original trial dataset, the absolute LOS difference between groups was extremely small (approximately 0.01 days) and is unlikely to represent a clinically meaningful difference in discharge timing or hospital resource utilization.

### 3.3. Secondary Outcomes

#### 3.3.1. Pain Intensity

Pain intensity was assessed using the Visual Analog Scale (VAS), which ranges from 0 (no pain) to 10 (worst imaginable pain). The pain score data were available for 41 participants in the IV acetaminophen group and 43 participants in the placebo group (see Table 3). During the first 24 h after surgery, the mean pain score in the acetaminophen group was 0.62 (IQR 0.13–1.00), whereas the placebo group reported 0.88 (IQR 0.38–2.00). Although pain scores numerically favored IV acetaminophen, the differences were not statistically significant and should therefore be interpreted cautiously, particularly given the modest sample size of the original trial.

#### 3.3.2. Opioid Consumption

The postoperative opioid requirement was measured in morphine milligram equivalents (MME) over a 24 h period. Participants in the acetaminophen group required a median of 42 MME (IQR 36.4–50.0), compared with 50 MME (IQR 35.6–72.0) in the placebo group. Although postoperative opioid consumption numerically favored IV acetaminophen, the difference did not reach statistical significance and may have been influenced by limited statistical power and perioperative multimodal analgesic co-interventions.

### 3.4. Adverse Events and Safety Outcomes

All participants were monitored for 30 days following surgery to evaluate safety and potential adverse effects. Importantly, there were no serious or non-serious adverse events reported in either the acetaminophen or placebo group, and no deaths occurred throughout the observation period. A summary of postoperative outcomes and safety measures comparing IV acetaminophen with placebo is presented in Table 4.

## 4. Discussion

### 4.1. Efficacy and Economic Impact of Intravenous Acetaminophen on Length of Stay (LOS)

The present secondary descriptive analysis of publicly available randomized trial data suggests that IV acetaminophen may contribute to modest improvements in perioperative recovery parameters when incorporated into multimodal analgesic protocols. Although statistical significance for hospital LOS was reported in the original trial dataset, the absolute difference between groups was extremely small and likely of limited clinical relevance. Similarly, numerical trends toward lower pain scores, opioid consumption, and shorter PACU stay favored IV acetaminophen but did not consistently achieve statistical significance. Importantly, no adverse or serious events were reported, supporting the overall safety and tolerability of repeated perioperative IV acetaminophen administration in this surgical setting. Patients who received IV acetaminophen had a median LOS of 0.81 days compared with 0.82 days in the placebo group (*p* = 0.006). Although differences in postoperative pain scores and opioid use were not statistically significant, numerical trends favored IV acetaminophen, and the shorter PACU stay may reflect a trend toward improved early postoperative recovery parameters.

These findings are generally consistent with large-scale economic and clinical analyses that have reported potential operational and economic advantages associated with IV acetaminophen use [19,20,21,22,23,24,25,26,27,28] (see Table 5). For example, Shaffer et al. [21] analyzed over 2.2 million inpatient encounters from 297 hospitals and modeled the effect of reducing opioid exposure while adding IV acetaminophen. Their analysis estimated an average 18.5% reduction in LOS, ranging from 10.7 to 32% depending on procedure type, and a 28.7% reduction in opioid-related complications. The modeled impact corresponded to approximately USD 4.5 million in LOS-related savings and total projected annual savings of USD 4.7 million for a medium-sized hospital. Although the study was not a randomized trial, its large sample size and strong associations suggest that IV acetaminophen may contribute to reduced LOS and lower inpatient costs in selected surgical settings; however, the magnitude of LOS reduction observed in our dataset was substantially smaller and should be interpreted cautiously [21].

Chidambaran et al. [22] extended this evidence through a prospective and model-based evaluation in adolescents undergoing spinal fusion. The combination of IV acetaminophen and ketorolac significantly reduced opioid consumption, improved gastrointestinal tolerance, and shortened LOS by approximately 0.5 days. In their decision-analytic model, the multimodal approach combining opioids, IV acetaminophen, and ketorolac was both the least costly and the most effective strategy, yielding cost savings of USD 510 to 947 per patient. This supports the potential utility of IV acetaminophen as an opioid-sparing adjunct within multimodal analgesia strategies, which may contribute to improved perioperative recovery parameters and shorter hospital stays without apparent safety concerns [22].

Similarly, Patel et al. [23] conducted a retrospective cohort study involving 199 adults to evaluate whether the addition of IV acetaminophen (Ofirmev) to standard pain management with opioids and ketorolac could improve recovery outcomes. Among these patients, 101 received IV acetaminophen in combination with opioids and ketorolac, while 98 received the standard regimen without IV acetaminophen. The results demonstrated that patients treated with IV acetaminophen had significantly shorter hospital and ICU stays, as well as reduced time to extubation, compared with those who received conventional pain management. For instance, the average hospital stay decreased from 2.95 to 2.33 days, and the time to extubation was notably shorter [23].

A similar cost advantage was observed by Maiese et al. [24] in an analysis of more than 140,000 orthopedic procedures using the Truven Health MarketScan Hospital Drug Database. Patients who received multimodal regimens including IV acetaminophen incurred mean total hospitalization costs of USD 12,540 compared with USD 13,242 for those treated with IV opioid monotherapy (*p* < 0.0001). After adjustment for confounders, the IV acetaminophen group still demonstrated a cost reduction of approximately USD 830 per patient, driven mainly by lower room and board expenses. These findings reinforce that IV acetaminophen, even when pharmacy costs are similar, may be associated with downstream operational and economic advantages by shortening LOS and improving postoperative efficiency, which is consistent with the shorter hospital stay observed in our study [24].

In pediatric populations, Mahdi et al. [25] investigated the impact of IV acetaminophen pricing on utilization, opioid consumption, and pharmacy costs in children undergoing appendectomy. Despite a substantial price increase in 2014, IV acetaminophen use increased from 3% in 2011 to 40.1% in 2017, and adjusted median pharmacy charges decreased from USD 3326.5 to USD 3264.1 (*p* < 0.001). Postoperative opioid use declined from 73.6% to 58.6%, suggesting that potential operational and resource-utilization advantages may persist despite higher drug costs, primarily through reduced opioid-related expenditures. These findings parallel our observations, indicating that IV acetaminophen may reduce hospital resource utilization even in populations with low baseline opioid use [25].

Consistent results have also been reported across large-scale real-world datasets by Hansen and colleagues [26,27,28]. In a 2018 analysis of 22,828 hysterectomy patients, those who received IV acetaminophen had hospital stays 0.8 days shorter and total costs USD 2449 lower than those receiving oral acetaminophen [26]. In a 2017 study involving 112,586 spine surgery patients, IV acetaminophen reduced LOS by 0.68 days and hospitalization costs by USD 1175 while lowering opioid requirements and reducing the need for post-acute facility care [27]. Their earlier 2016 study in 485,895 orthopedic procedures reported similar findings, where adding IV acetaminophen reduced LOS by 0.51 days, decreased hospitalization costs by USD 635, and lowered rates of urinary and respiratory complications [28]. Across these diverse surgical populations, consistent reductions in LOS from 0.5 to 0.8 days and cost savings ranging from USD 600 to 2400 per patient suggest potentially reproducible operational and economic advantages across diverse surgical populations of IV acetaminophen compared with opioid monotherapy or oral formulations [26,27,28]. These findings emphasize the importance of distinguishing statistical significance from clinical relevance, particularly when absolute differences in LOS are minimal.

### 4.2. Timing-Dependent Analgesic Efficacy and Pain Relief Outcomes

In this study, patients receiving IV acetaminophen, OFIRMEV, reported lower mean pain scores during the first 24 h (0.62 vs. 0.88) and the second 24 h (1.28 vs. 2.25) postoperatively compared with placebo, indicating a trend toward improved analgesia. Although these differences did not reach statistical significance, the trend suggests that IV contributes to improved pain control when used as part of a multimodal analgesic regimen.

These findings are consistent with Strode et al. [29], who conducted a randomized, double-blind, placebo-controlled study in 34 patients undergoing laparoscopic sleeve gastrectomy. The results showed that patients treated with OFIRMEV experienced significantly lower pain scores at 12, 16, and 20 h after surgery compared to their baseline, while the placebo group did not show any meaningful improvement. Altogether, the study demonstrated that IV OFIRMEV may reduce postoperative pain perception, even if it does not necessarily decrease opioid use within the first 24 h.

Conversely, the analgesic effect of IV acetaminophen is not consistent across all surgical contexts. Atencio et al. [30] evaluated 72 patients undergoing extraction of partially or fully impacted third molars. Pain scores at immediate, 4 h, and 24 h postoperative intervals showed no statistically significant difference between the IV acetaminophen and placebo groups. The lack of detectable analgesic benefit was likely due to low baseline pain levels and the use of local anesthetics, which may have masked the incremental effect of IV acetaminophen.

Timing of administration appears to play a pivotal role in optimizing the analgesic efficacy of IV acetaminophen. Atencio et al. [30] further reviewed multiple surgical settings, demonstrating that preemptive administration prior to surgical incision significantly reduced postoperative pain scores, while intraoperative dosing enhanced recovery metrics such as earlier discontinuation of patient-controlled analgesia (PCA). Postoperative administration in the PACU also improved pain scores in high-intensity procedures like total knee arthroplasty. These findings suggest that the maximal analgesic effect of IV acetaminophen is achieved when administered early in the perioperative period, which may explain why pain reductions in our study were more pronounced in the first 24 h [30].

Elvir-Lazo et al. [31] reinforced this concept, showing that preemptive or intraoperative IV acetaminophen provides the most substantial reductions in postoperative pain and opioid requirements, whereas delayed postoperative dosing yields more modest benefits. In the referenced clinical trial (NCT02369211) [18], pain scores in the IV acetaminophen group remained lower than those in the control group during the first 24 h following surgery and gradually converged with control scores during the subsequent 24 h. This pattern aligns with the observations of Elvir-Lazo et al. [31], supporting the notion that early administration of IV acetaminophen may enhance analgesic effectiveness and may contribute to postoperative recovery.

Pediatric studies similarly reflect this nuanced effect. Lammers et al. [32] compared a single oral acetaminophen loading dose (30 mg/kg) to IV acetaminophen (15 mg/kg) in 66 children undergoing tonsillectomy and adenoidectomy. Pain scores and opioid consumption in the first 24 h postoperatively were not statistically different, likely due to standardized multimodal analgesia effectively controlling pain in both groups. Similarly, Sola Jr et al [33] reported no significant differences in pain scores or Patient-Controlled Analgesia (PCA) opioid use in children with perforated appendicitis receiving IV acetaminophen, suggesting that robust opioid regimens may mask additional analgesic effects of IV acetaminophen.

Finally, Luo and Min [34] highlighted that multimodal analgesia, including IV nonopioid agents such as acetaminophen, can improve pain control in the postanesthesia care unit (PACU), though efficacy depends on the timing of administration, surgical pain severity, and concurrent analgesic techniques. They suggested that objective pain assessments, such as pupillometry or skin conductance, may better capture analgesic benefits in complex surgical cases.

### 4.3. Opioid-Sparing Effects of Intravenous Acetaminophen

Administration of IV acetaminophen was associated with numerically lower postoperative opioid consumption in the original trial dataset, consistent with previously discussed findings. This observation aligns with several large-scale and multicenter studies evaluating the opioid-sparing effects of IV acetaminophen in both pediatric and adult populations. For example, Shaffer et al. [21] analyzed inpatient encounters from 297 hospitals to model the impact of IV acetaminophen on opioid use. Their model demonstrated that the addition of IV acetaminophen combined with a reduction in opioid use by one level (high → medium, medium → low, low → none) led to a 28.7% decrease in opioid-related complications. This model may contribute to lower opioid requirements while simultaneously contributing to reduced length of stay and inpatient costs.

Similarly, Chidambaran et al. [22] further reported notable reductions in opioid requirements following the introduction of IV acetaminophen, with corresponding improvements in comfort and recovery. In large-scale data, Mahdi et al. [25] documented a substantial decline in opioid use from 73.6% to 58.6% as IV acetaminophen utilization increased from 3% to 40.1%. Additionally, Luo and Min [34] found that an IV acetaminophen–opioid combination was both less costly and more effective, requiring fewer rescue doses in pediatric tonsillectomy cases. Altogether, these studies support the potential opioid-sparing role of IV acetaminophen and suggest that even incremental reductions in opioid exposure can have cumulative clinical and safety benefits.

In a focused study on infants under 1 year undergoing abdominal or anorectal surgery, Vavolizza et al. [35] implemented a standardized IV acetaminophen protocol administered every six hours for a minimum of 48 h. This intervention led to a 36-fold reduction in postoperative morphine equivalents (median 0.36 mg/kg pre-intervention vs. 0 mg/kg post-intervention, *p* < 0.0001), without any compromise in pain scores measured by the FLACC scale. These results provide strong evidence that IV acetaminophen can achieve profound opioid sparing in high-risk pediatric populations, corroborating the trends observed in our cohort.

Archer et al. [36] conducted a systematic review and meta-analysis of five randomized controlled trials including 443 pediatric patients undergoing major abdominal and thoracic surgeries. The analysis revealed that adding IV acetaminophen to opioid-based analgesia resulted in a reduction in opioid consumption (−1.95 morphine equivalents/kg/48 h), while maintaining equivalent pain scores compared with opioids alone. Although the overall certainty of evidence was rated as low, the findings suggest that IV acetaminophen effectively decreases opioid requirements and minor adverse events, supporting its role as a valuable component of multimodal analgesia.

Altogether, the cumulative evidence is generally consistent with the numerical trends observed in our secondary descriptive analysis, suggesting that IV acetaminophen may contribute to reduced postoperative opioid requirements in selected perioperative settings. The extent of this reduction depends on factors such as how painful the procedure is, when the medication is given, and what other pain treatments are used alongside it. Using IV acetaminophen as part of a multimodal pain management plan may represent a useful adjunctive strategy within multimodal analgesia protocols aimed at limiting opioid exposure and supporting postoperative recovery.

## 5. Limitations

Several limitations should be acknowledged when interpreting the findings of this secondary descriptive analysis. First, all analyzed data were derived from publicly available aggregate trial results rather than individual patient-level datasets, limiting the ability to independently verify statistical analyses, assess covariate balance, perform adjusted analyses, or calculate additional effect size estimates.

Second, the original randomized trial was conducted at a single tertiary center with a relatively modest sample size, which may limit external validity and reduce statistical power to detect small differences in perioperative outcomes, particularly for secondary endpoints.

Third, differing participant numbers across outcome analyses indicate incomplete or missing registry-reported outcome data, which may introduce attrition bias and affect internal comparability between groups. Because the publicly available registry materials did not provide detailed characteristics of participants with incomplete outcome data, the direction and magnitude of potential attrition bias could not be independently assessed.

Fourth, although statistical significance for hospital length of stay was reported in the original trial dataset, the absolute difference between groups was extremely small and unlikely to represent a clinically meaningful improvement in discharge timing or hospital resource utilization. Interpretation should therefore distinguish statistical significance from practical clinical relevance.

Fifth, effect size estimates and confidence intervals for several secondary outcomes were not consistently available within the publicly accessible registry materials, limiting more detailed comparative interpretation.

Importantly, no formal pharmacoeconomic, cost-minimization, or cost-effectiveness analysis was conducted in either the original trial or the present secondary descriptive analysis. Therefore, the economic implications discussed in this manuscript should be interpreted as exploratory and hypothesis-generating rather than definitive economic conclusions.

Additionally, variability in perioperative protocols, multimodal analgesic co-interventions, and timing of intravenous acetaminophen administration may have influenced pain and opioid-related outcomes. Finally, longer-term endpoints such as readmission rates, functional recovery, patient-reported outcomes, and post-discharge healthcare utilization were not available for evaluation.

## 6. Conclusions

This secondary descriptive analysis of publicly available randomized trial data suggests that multi-dose intravenous acetaminophen may contribute to modest improvements in perioperative recovery parameters when incorporated into multimodal analgesia protocols for robotic-assisted laparoscopic prostatectomy. Although numerical trends favored intravenous acetaminophen for pain scores, opioid consumption, and recovery outcomes, most differences were not statistically significant, and the observed reduction in hospital LOS was clinically minimal despite statistical significance reported in the original trial dataset.

Nevertheless, intravenous acetaminophen appeared safe and well-tolerated, with no reported serious adverse events. The findings support continued investigation of intravenous acetaminophen as a potentially useful adjunct within multimodal perioperative analgesia strategies; however, larger multicenter studies incorporating formal economic analyses and long-term outcome assessment are needed before definitive clinical or pharmacoeconomic conclusions can be established.

## Figures and Tables

**Table 1:** Baseline Characteristics of Participants in the Intravenous Acetaminophen and Placebo Groups.

Characteristic	Intravenous Acetaminophen (1 g IV) (n = 43)	Placebo (Saline) (n = 41)	Total (n = 84)
Participants started (n)	43	43	86
Participants completed (included in analysis) (n)	43	41	84
Participants not completed, (n)	0	2	2
Age, categorical (years), n (%)			
18–65 years	14 (32.6%)	16 (39.0%)	30 (35.7%)
≥65 years	29 (67.4%)	25 (61.0%)	54 (64.3%)
Age, continuous (years)			
Mean ± SD (years)	63.6 ± 6.1	60.2 ± 6.3	61.7 ± 6.0
Sex, n (%)			
Female	0 (0.0%)	0 (0.0%)	0 (0.0%)
Male	43 (100.0%)	41 (100.0%)	84 (100.0%)
Race and ethnicity			
Race and ethnicity collected	Not reported	Not reported	Not reported
Region of enrollment, n			
United States	43	41	84

**Table 2:** Primary Outcomes.

Outcome	Intravenous Acetaminophen (1 g IV)	Placebo (Saline)
Post-anesthesia care unit (PACU) length of stay		
Number analyzed	34	41
Mean ± SD	124 ± 58.26	132 ± 62.58
Hospital length of stay		
Number analyzed	34	41
Median [95% CI]	0.81 [0.71–0.90]	0.82 [0.66–0.95]

**Table 3:** Secondary Outcomes.

Outcome	Intravenous Acetaminophen (1 g IV)	Placebo (Saline)
**Pain score (0–24 h after surgery)**		
Scale description	Visual Analog Scale (0 = no pain, 10 = worst pain)	
Number analyzed	41	43
Median [IQR]	0.62 [0.13–1.00]	0.88 [0.38–2.00]
**Opioid use (0–24 h after surgery)**		
Number analyzed	43	41
Median [IQR]	42.0 [36.4–50.0]	50.0 [35.6–72.0]

Note: Median (Inter-Quartile Range) = *IQR*.

**Table 4:** Postoperative Outcomes and Safety Measures in Patients Receiving Intravenous Acetaminophen versus Placebo.

Outcome Measure	Type/Time Frame	Description
Post-anesthesia care unit (PACU) length of stay	Primary/~30–240 min	Time patients spent in the post-anesthesia care unit following surgery and anesthesia before transfer to the inpatient ward (immediate recovery).
Hospital length of stay	Primary/1–3 days	Total number of days the patient remained hospitalized from surgery until discharge. Shorter stays may reflect improved recovery efficiency and reduced hospital resource utilization.
Pain score (Visual Analog Scale, 0–10)	Secondary/0–24 h post-surgery	Patient-reported pain intensity during the first 24 h postoperatively, using a 0–10 scale (0 = no pain, 10 = worst pain).
Opioid use (morphine milligram equivalents, MME)	Secondary/0–24 h	Total opioid consumption during the first 24 h after surgery, expressed as morphine milligram equivalents (mg MME).
All-cause mortality	Safety/Follow-up: 30 days	Number of deaths from any cause within 30 days after surgery.
Serious adverse events	Safety/Follow-up: 30 days	Events resulting in death, life-threatening conditions, new or prolonged hospitalization, or persistent/significant disability.
Other (non-serious) adverse events	Safety/Follow-up: 30 days	Non-serious adverse events, including minor complications such as nausea, mild allergic reactions, or local injection-site reactions.

**Table 5:** Summary of economic and Clinical Evidence Supporting Intravenous Acetaminophen (OFIRMEV) in Perioperative Care.

Ref.	Surgical Population/Setting	Study Type	Comparison/Intervention	Key Economic or Clinical Outcomes
Shaffer et al. [21]	Mixed inpatient surgeries (2.2 million encounters, 297 hospitals)	Retrospective modeling analysis	IV acetaminophen + reduced opioid exposure vs. opioid monotherapy	Average 18.5% reduction in length of stay (LOS) (range 10.7–32%) and 28.7% reduction in opioid-related complications; projected USD 4.7 million annual hospital savings.
Chidambaran et al. [22]	Adolescent spinal fusion	Prospective cohort + decision-analytic model	IV acetaminophen + ketorolac vs. opioid alone	LOS shortened by ≈0.5 days; improved gastrointestinal tolerance; cost savings USD 510–947 per patient; lowest cost and highest effectiveness among compared regimens.
Patel et al. [23]	Adult thoracic surgery (199 patients)	Retrospective cohort	IV acetaminophen + opioid + ketorolac vs. standard regimen (opioid + ketorolac)	LOS decreased from 2.95 to 2.33 days; shorter ICU stay; reduced time to extubation; improved recovery efficiency.
Maiese et al. [24]	Orthopedic procedures (>140,000 cases)	Retrospective database (Truven Health MarketScan)	IV acetaminophen multimodal regimen vs. IV opioid monotherapy	Mean hospitalization cost USD 12,540 vs. USD 13,242 (*p* < 0.0001); adjusted USD 830 cost reduction per patient, driven by shorter LOS and lower room/board charges.
Mahdi et al. [25]	Pediatric appendectomy (2011–2017)	Retrospective trend analysis	Before vs. after IV acetaminophen implementation	IV acetaminophen use increased from 3% → 40.1%; opioid use declined 73.6% → 58.6%; median pharmacy cost decreased from USD 3326.5 → 3264.1 (*p* < 0.001).
Hansen et al. [26]	Hysterectomy (22,828 patients)	Retrospective analysis	IV acetaminophen vs. oral acetaminophen	LOS reduced by 0.8 days; total cost lower by USD 2449; fewer complications.
Hansen et al. [27]	Spine surgery (112,586 patients)	Retrospective analysis	IV acetaminophen vs. oral acetaminophen	LOS decreased by 0.68 days; cost reduced by USD 1175; fewer readmissions and lower post-acute facility use.
Hansen et al. [28]	Orthopedic procedures (485,895 patients)	Retrospective analysis	IV acetaminophen + opioids vs. IV opioids alone	LOS shorter by 0.51 days; cost savings ≈USD 635; lower urinary and respiratory complication rates.

## Data Availability

The data presented in this study are available in ClinicalTrials.gov at https://clinicaltrials.gov/study/NCT02369211?term=NCT02369211&rank=1 [18] (accessed on 4 April 2026).
